# Effects of Complete Submergence on Growth, Survival and Recovery Growth of *Alisma orientale* (Samuel.) Juz.

**DOI:** 10.3390/plants13223189

**Published:** 2024-11-13

**Authors:** Songping Liu, Jingrui Liu, Feng Lin, Libing Liao, Qian Hu, Lei Xu, Ludan Chen, Te Cao, Aiwen Zhong

**Affiliations:** 1 Jiangxi Province Key Laboratory of Wetland Plant Resources Conservation and Utilization, Lushan Botanical Garden, Jiangxi Province and Chinese Academy of Sciences, Jiujiang 332900, China; lsp852319@163.com (S.L.); ljr1321676627@163.com (J.L.); liaolb@lsbg.cn (L.L.); abi10qhu@hotmail.com (Q.H.); xul@lsbg.cn (L.X.); chenld@lsbg.cn (L.C.); 2 School of Life Sciences, Nanchang University, Nanchang 330031, China; 3 Key Laboratory of Eco-Environments in Three Gorges Reservoir Region (Ministry of Education), Chongqing Key Laboratory of Plant Ecology and Resources in Three Gorges Reservoir Region, School of Life Sciences, Southwest University, Chongqing 400715, China; linfable@swu.edu.cn; 4 Donghu Experimental Station of Lake Ecosystems, Institute of Hydrobiology, Chinese Academy of Sciences, Wuhan 430072, China

**Keywords:** complete submergence, elongation, survival, recovery growth, *Alisma orientale*

## Abstract

Intense precipitations caused by global climate change will result in the occurrence of greater frequencies and longer durations of flooding, influencing the survival and yields of wetland plants. *Alisma orientale* (Samuel.) Juz., an important traditional medicine with edible scape and inflorescence, naturally grows in wetlands and artificially cultivates in paddy fields prone to flood in China. However, we lack understanding of the effect of complete submergence on *A*. *orientale*. Here, experiments with four durations of complete submergence including 5 days (ds), 10 ds, 15 ds and 20 ds followed by 20 ds recovery were performed. In the submergence experiments, the number of, length of and biomass of surviving leaves and the total biomass and new blade biomass were measured; in recovery experiments, number and length of surviving leaves were measured. *A. orientale* grew out longer new leaves during complete submergence, with a dramatic decline in the biomass of both the leaves and tubers as well as the total biomass at the ends of the submergence experiments. The *A. orientale* plants had a high survival rate after submergence. The duration of submergence did not influence the time for *A. orientale* needed to start regrowing. At the end of recovery period, the submerged *A. orientale* plants generated more leaves, had more surviving leaves, had shorter new leaves and a shorter total length of surviving leaves than the control plants. This study highlights that *A. orientale* plants can resist at least 20 ds of complete submergence caused by flooding and regrow rapidly after submergence and improves our understanding of the flooding tolerance mechanisms of *A. orientale* plants.

## 1. Introduction

Flooding is an important environment factor influencing the survival, distribution and productivity of plants in worldwide wetlands and paddy fields. In these areas, the number of flood events will rise in the near future [1,2], due to increases in the magnitudes and frequencies of heavy rainfall induced by global climate change [3,4]. Owing to flooding, plants growing in these areas are often subjected to waterlogging and even complete submergence [5,6,7]. Greater frequencies and longer durations of flooding stress, especially complete submergence, will be imposed on these plant species in the era of global warming, resulting in crop yield reductions and, subsequently, huge economic losses [8,9].

Complete submergence due to flooding results in profound changes in the surrounding environments of plants distributed in flood-prone habitats [5,6,7,10]. During submergence, one of these changes is poor light transmission in a flood–water column, which depends on turbidity of flood water [11], weakening photosynthesis dramatically [7,12,13]. Additionally, photosynthesis is also limited by underwater carbon supply [5,7], due to low gas diffusion in water [7], although some species can be capable of photosynthesis underwater [7,12,13]. Due to the low gas diffusion and, more importantly, hypoxia, anaerobic processes occur in the whole plant, not only in the submerged plant roots [7,14]. Hypoxia or anaerobic conditions lead to the metabolism of ethanol and an accelerated consumption of non-structural carbohydrates, eventually resulting in a reduction of biomass in a flooded plant [15]. Meanwhile, reactive oxygen species (ROS) gradually accumulate in its submerged organs due to hypoxia [10,15], and ROS results in post-anoxic injury when the flood recedes, ultimately diminishing plant survival and crop yield [10,15].

To cope with complete submergence, plant species inhabiting permanently or temporarily flooded areas have evolved two contrasting strategies: the quiescence and escape strategies [16,17]. Some plant species employ a quiescence strategy in which growth and metabolism are low, even repressed under submergence conditions [6,17]. Conversely, some species confront submergence with an escape strategy through the elongation of internodes or petioles, which can restore contract with air [16,17]. Elongation is costly, resulting in the decline of carbohydrate reserves and biomass, even influencing survival [6,18,19]. Regardless of which strategy the inundated species employ, flood-tolerant plant species can revive rapidly, with relatively high survival during post-submergence after the deluge [10,20].

Our understandings of flooding responses and flooding-tolerance mechanisms originate mainly from model species of *Oryza* and *Arabidopsis* species [18,19,21,22], while abundant studies from some wild plant species, such as *Rumex* species [13,23], the genus *Ranunculus* [17], *Rorippa* spp. [24], *Alternanthera philoxeroides* (Mart.) Griseb. [25,26] and *Cynodon dactylon* (L.) Persoon [27], also adds many unprecedented insights into the mechanisms of flooding tolerance [6]. Thus, some unidentified genes and processes conferring flooding tolerance [28] will be found in other wild or less-discussed species, for example species with medicinal value and edibility [24,27,29,30,31,32,33], although these species have received little, if any, attention.

*Alisma orientale* (Samuel.) Juz. Is a perennially aquatic or marsh herb distributed widely in China [34]. *A. orientale* also is a sympodial semirosellate herb [35] consisting of roots, tuber and leaves (Figure S1A) [35]. The tubers of *A. orientale* (Figure S1B) are a well-known and an important traditional Chinese medicine in East Asia [36,37,38], especially in China; its immature scape and inflorescence (Huatai in Chinese, 花薹; Figure S1C) are health-promoting vegetables [36]. In China, *A. orientale* plants have two growing seasons: one growing season ranges from March to June, where it gains its scape and inflorescence, whereas the other ranges from August to December, where it gains its scape and inflorescence and, chiefly, its tuber.

In *Alisma* species, the knowledge regarding the suites of traits adapted to aquatic environments (waterlogging and partial submergence) mainly comes from *A. plantago-aquatica* [29,32,33,39,40,41] and marginally from *A. triviale*, *A. canaliculatum* and *A. orientale* [30,31]. Regarding species of *Alisma*, although botany textbooks by Sculthorpe have recorded that they can grow in complete submergence conditions [42], they were still influenced by complete submergence. For all we know, we have little basic knowledge of the impacts of complete submergence on the *A. orientale* plants. We, therefore, conducted two experiments lasting 20 days for *A. orientale*: one is an experiment on complete submergence, the other is an experiment on post-submergence recovery. The three specific questions addressed in this paper are: (1) Which strategy do *A. orientale* plants adopt when subjected to complete submergence? (2) Do *A. orientale* plants have a high survival rate? (3) Can *A. orientale* plants regrow rapidly?

## 2. Results

### 2.1. Growth Response During Complete Submergence

**Figure 1:**
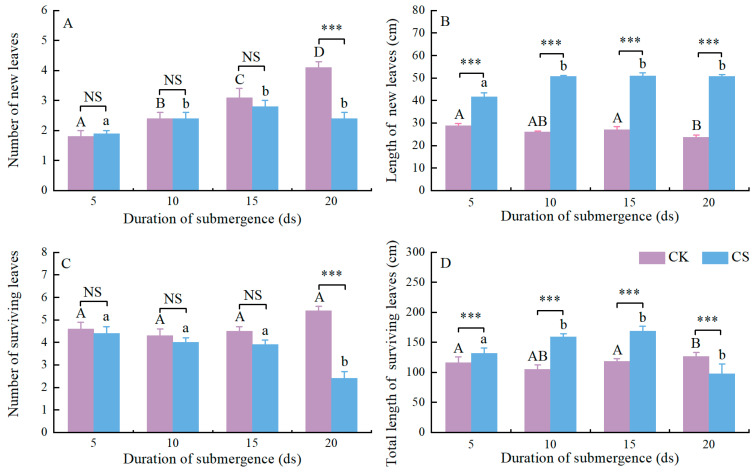
Growth response of *Alisma orientale* (Samuel.) Juz. plants after four different durations of complete submergence. (**A**) Number of new leaves. (**B**) Length of a new leaves. (**C**) Number of surviving leaves. (**D**) Total length of surviving leaves. Within control treatments (CK), values with different capital letters are significantly different using one-way ANOVA followed by the Duncan’s multiple-range tests. Within submergence treatments (CS), values with different lowercase letters are significantly different using one-way ANOVA followed by the Duncan’s multiple-range tests. NS, not significantly different; ***, *p* < 0.001; independent-samples *t*-tests. Data are means ± SE. Number of replicates is eight.

An increased number of new leaves occurred in the *A. orientale* plants during the first 15 days (ds) of complete submergence; this did not occur after 15 ds of submergence (Figure 1A). There were no differences in the numbers of new leaves for *A. orientale* between plants with no submergence and those that were submerged for 5 ds, 10 ds and 15 ds; however, the numbers of new leaves were significantly less (*p* < 0.001) in the plants with 20 ds of submergence than those in the plants without submergence (Figure 1A). The lengths of new leaves were significantly longer in a submerged plant than in a control plant in the four submergence treatments (plants submerged for 5 ds, *p* < 0.001; for 10 ds, *p* < 0.001; for 15 ds, *p* < 0.001; for 20 ds, *p* < 0.001; Figure 1B and Figure S2). The lengths of the new leaves elongated to a maximum at 10 d (Figure 1B). There were no differences in the numbers of surviving leaves in *A. orientale* plants between the control and submerged plants in the three shortest submergence treatments (plants submerged for 5 ds, *p* > 0.050; for 10 ds, *p* > 0.050; for 15 ds, *p* > 0.050; Figure 1C), but the number of surviving leaves was significantly higher in a plant with 20 ds of submergence than that in a plant without submergence (*p* < 0.001; Figure 1C). The number of surviving leaves tended to gradually decline (Figure 1C). There were no differences in the total lengths of surviving leaves for *A. orientale* pants between the submergence treatment and the control after 5 ds of submergence, as well as after 20 ds of submergence, but the total length of the surviving leaves for *A. orientale* were significantly longer in the submerged plants than in the controls after 10 ds of submergence (*p* < 0.001), as well as after 15 ds of submergence (*p* < 0.001) (Figure 1D). The increased total length of surviving leaves in *A. orientale* occurred in the first 15 ds of submergence and did not after 15 ds of submergence (Figure 1D).

### 2.2. Biomass Loss During Complete Submergence

**Figure 2:**
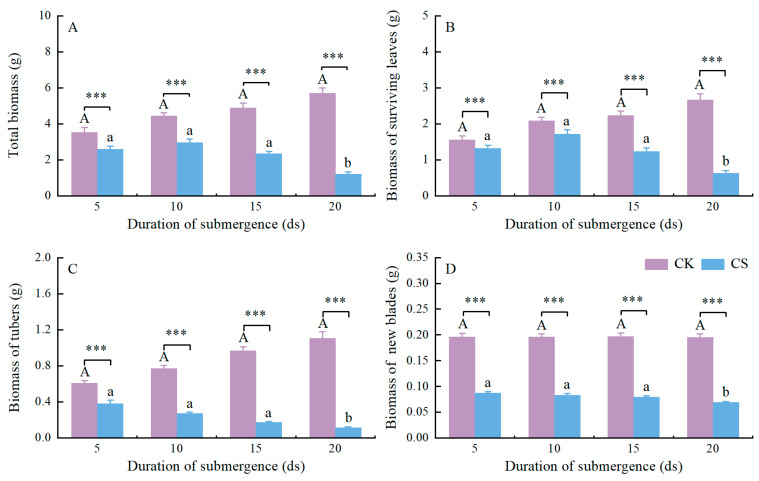
Biomass of *A. orientale* plants after four different durations of complete submergence. (**A**) Total biomass. (**B**) Biomass of surviving leaves. (**C**) Biomass of tubers. (**D**) Biomass of a new blades. Within control treatments (CK), values with different capital letters are significantly different (*p* < 0.050, one-way ANOVA followed by the Duncan’s multiple-range tests). Within submergence treatments (CS), values with different lowercase letters are significantly different (*p* < 0.050, one-way ANOVA followed by the Duncan’s multiple-range tests). Significant differences between submerged plants and control plants after each duration of submergence are indicated: ***, *p* < 0.001 (independent-sample *t*-tests). Data are means ± SE. Number of replicates is eight.

The total biomass for *A. orientale* was remarkably less in a submerged plant than in a control plant in all complete submergence treatments (plants submerged for 5 ds, *p* < 0.001; for 10 ds, *p* < 0.001; for 15 ds, *p* < 0.001; for 20 ds, *p* < 0.001; Figure 2A). The total biomass of *A. orientale* plants reduced with the duration of submergence (Figure 2A). The biomass of surviving leaves for *A. orientale* was remarkably less in the submerged plants than that in the controls in these three treatments (plants submerged for 10 ds, *p* = 0.033; for 15 ds, *p* < 0.001; for 20 ds, *p* < 0.001; Figure 2B), except there were no differences between the submerged plants and the controls after 5 ds of submergence (*p* > 0.050; Figure 2B). The biomass of surviving leaves in the *A. orientale* plants had a slight increase in the first 5 ds of complete submergence but reduced after 10 ds of submergence (Figure 2B). The tuber biomass was remarkably less in a submergence plant than in a control plant in all treatments (plants submerged for 5 ds, *p* < 0.001; for 10 ds, *p* < 0.001; for 15 ds, *p* < 0.001; for 20 ds, *p* < 0.001; Figure 2C). The biomass of tubers in the *A. orientale* plants always declined during submergence (Figure 2C). The biomass of new leaves was remarkably less in the submerged plants than that in the controls in all treatments (plants submerged for 5 ds, *p* < 0.001; for 10 ds, *p* < 0.001; for 15 ds, *p* < 0.001; for 20 ds, *p* < 0.001; Figure 2D). The biomass of new leaves in the *A. orientale* plants also declined during submergence (Figure 2D).

### 2.3. Survial Rate at the End of the Submergence and Recovery Periods

All the control plants had survived at the ends of both the submergence and recovery periods (Table 1). At the end of each submergence period, the survival of *A. orientale* plants was 100% in all submergence treatments (plants had been submerged for 5 ds, for 10 ds, for 15 ds and for 20 ds; Table 1). At the ends of the recovery periods, the survival of *A. orientale* plants was still 100% in three of the recovery treatments (plants had been submerged for 5 ds, for 10 ds and for 15 ds; Table 1); it was 87.5% in the recovery treatment after 20 ds of submergence (Table 1).

### 2.4. Recovery Growth During Post-Submergence

**Figure 3:**
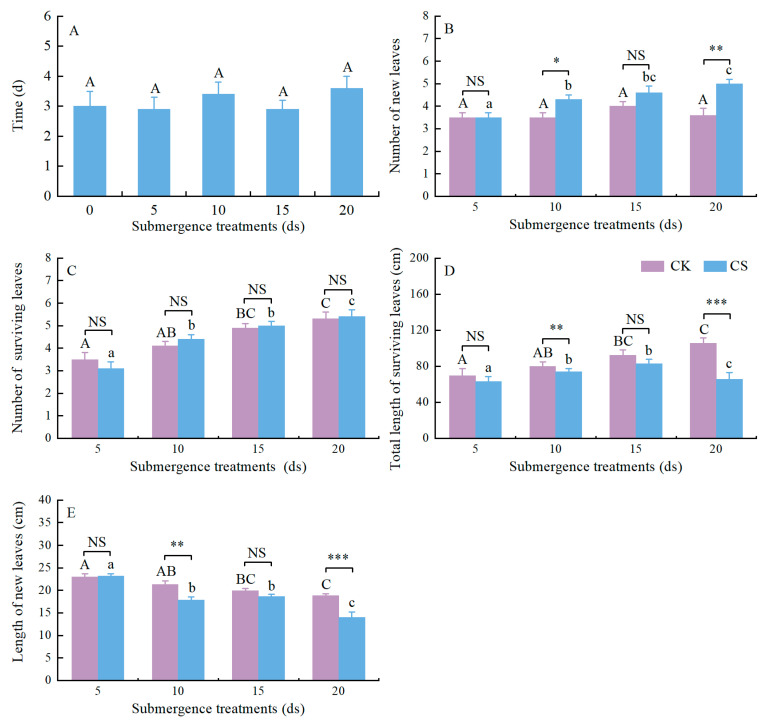
Recovery growth of the *A. orientale* plants following complete submergence for four different durations. (**A**) The time that *A. orientale* needed to start recovery growth. (**B**) The number of new leaves within 20 ds following complete submergence. (**C**) The number of surviving leaves at the end of complete submergence. (**D**) The length of a new leaf at the end of complete submergence. (**E**) The total length of surviving leaves at the end of complete submergence. In the data for the lengths of time that plants needed to regrow during the recovery periods, the same letters above the bars indicate significant differences at *p* > 0.050 using one-way ANOVA followed by Duncan tests for multiple comparison. Within control treatments (CK), values with different capital letters are significantly different (*p* < 0.050, one-way ANOVA followed by Duncan tests for multiple comparisons). Within submergence treatments (CS), values with different lowercase letters are significantly different (*p* < 0.050, one-way ANOVA followed by Duncan tests for multiple comparisons). Significant differences between submerged plants and control plants within each recovery treatment are indicated at *p* > 0.050, NS; at *p* < 0.05, *; at *p* < 0.010, **; at *p* < 0.001, ***. (All data used independent-sample *t*-tests, except for the data on number of surviving leaves, which used Mann–Whitney U tests). Data are means ± SE. Number of replicates is eight, except for the plants submerged for 20 d (n = 7).

There were no significant differences among the lengths of time that the *A. orientale* plants needed to regrow following complete submergence (*F* = 0.566, *p* = 0.689; Figure 3A). The *A. orientale* plants displayed a strong recovery capability after complete submergence (Figures S2 and S4).

Within the 20 ds recovery periods following complete submergence, the numbers of new leaves for *A. orientale* were not significantly different in the plants submerged than in the control plants in two of the recovery treatments (plants followed by 5 ds of complete submergence, *p* > 0.050; by 15 ds of complete submergence, *p* > 0.050; Figure 3B), but were significantly different in the plants submerged than in the controls in the two other recovery treatments (plants followed by 10 ds of complete submergence, *p* = 0.015; by 20 ds of complete submergence, *p* = 0.005; Figure 3B). With an increase in the duration for which the plants were treated, the number of new leaves increased in the submerged plants (*F* = 9.023, *p* < 0.001; Figure 3B) but not in the controls (*F* = 1.270, *p* = 0.304; Figure 3B).

Significant differences in the numbers of surviving leaves between the submerged *A. orientale* plants and the controls were found in the recovery treatment after 10 ds of complete submergence with a 10 d recovery period (*p* = 0.006; Figure S4B) and in the treatment after 20 ds of complete submergence with the 5 d and 10 d recovery periods (plants with the 5 d recovery period, *p* = 0.001; with the 10 d, *p* = 0.016; Figure S4D) but not found in the other submergence treatments and other recovery periods (*p* > 0.050; Figure S4A–D). At the end of recovery period, the number of surviving leaves in the submerged plants was more than that in the controls in the all the submergence treatments (plants followed by 5 ds of complete submergence, *p* > 0.050; by 10 ds of complete submergence, *p* > 0.050; by 15 ds of complete submergence, *p* > 0.050; by 20 ds of complete submergence, *p* > 0.050; Figure 3C). With the increase in the duration that the plants had been treated, the number of surviving leaves increased in the submerged plants (*F* = 16.761, *p* < 0.001; Figure 3C) and also in the controls (*F* = 8.951, *p* < 0.001; Figure 3C).

Significant differences in the total length of the surviving leaves between the submerged *A. orientale* plants and the controls were always found in the recovery treatment after 20 ds of complete submergence during the whole recovery period (plants with a 5 d recovery period, *p* = 0.001; with 10 d, *p* = 0.016; with 15 d, *p* = 0.001; with 20 d, *p* = 0.016; Figure S4H). At the end of recovery period, significant differences in the total length of the surviving leaves in the *A. orientale* plants were not observed between the submerged plants and the controls in three recovery treatments (plants that had been submerged for 5 ds, *p* > 0.050; for 10 ds, *p* > 0.050; for 15 ds, *p* > 0.050; Figure 3D) but were observed in the recovery treatment after 20 ds of submergence (*p* = 0.001; Figure 3D). With the increase in the duration that the plants had been treated, the total length of surviving leaves in the controls increased dramatically (*F* = 6.424, *p* = 0.002; Figure 3D), but the total length of surviving leaves in the submerged plants increased dramatically earlier and decreased dramatically after 15 d of complete submergence (*F* = 2.990, *p* = 0.049; Figure 3D).

Significant differences in the leaf length between the submerged *A. orientale* plants and the controls were always found in the recovery treatment after 20 ds of complete submergence during the whole recovery period (plants with 5 d of recovery period, *p* = 0.001; with 10 d, *p* = 0.001; with 15 d, *p* = 0.001; with 20 d, *p* = 0.001; Figure S4L). At the end of recovery period, the lengths of the new leaves in the plants submerged for 5 ds were marginally longer than those in the controls (*p* > 0.050, Figure 3E); the lengths of the new leaves in the plants submerged for 15 ds were not strikingly longer than those in the controls (*p* > 0.050, Figure 3E); the length of the new leaves in both the plants submerged for 10 ds and for 20 ds were strikingly longer than those in the controls (plants followed by 10 ds of complete submergence, *p* = 0.003; by 20 ds, *p* = 0.001; Figure 3E). With the increase in the duration for which the plants had been treated, the length of the new leaves declined both in the submerged plants and in the controls (submergence, *F* = 26.211, *p* < 0.001; control, *F* = 8.609, *p* < 0.001; Figure 3E).

## 3. Discussion

Some plant species exposed to complete submergence environments can improve their survival by the elongation of nodes or petioles [6,7]. Elongation is beneficial for leaf blades’ contacts with the air [18,19,21,25,26]. In this study, it is clearly shown that complete submergence induced leaf elongation in the *A. orientale* plants (Figure 1B and Figure S2), coinciding with previous studies [6,24,25,26,43,44]. This is one of reasons species of *Alisma* can survive complete submergence conditions, even in deep water [41]. Additionally, during submergence, submerged plants can also generate aquatic leaves beneficial for underwater photosynthesis [12,13,45]. Despite the lack of morphological, anatomical and biochemical traits in this study, a noticeable reduction in the biomass of new blades was observed in submerged plants (Figure 2D). Elongation growth, however, did not include more leaves in submerged plants than in controls, and the increase in new leaf number was also repressed after 15 ds of submergence (Figure 1A). Furthermore, underwater elongation is accompanied by a large consumption of carbohydrates [46,47], and the resulting consequence is fatal when elongation does not allow leaf blades above the water surface [43]. During the submergence, the leaves of *A. orientale* plants did not emerge from the water, so the number of surviving leaves reduced gradually (Figure 1C) and the total length of surviving leaves decreased significantly after 15 ds of submergence (Figure 1D). Due to the elongation of new leaves (Figure 1B), 20 ds of complete submergence induced many old leaves to be lost in the submerged plants (Figure 1C) but did not cause a significant reduction in the total length of surviving leaves, relative to the controls (Figure 1D). To the best of our knowledge, this paper is the first that reports flooding responses to complete submergence in *A. orientale*, an important traditional Chinese medicine herb with edible value.

During complete submergence, the biomass of a plant always decreases [15,27], except for plant species typically spending their entire life cycle underwater. Gradual declines in the total biomass, leaf biomass, and tuber biomass of submerged plants were clearly observed (Figure 2A–C). A reasonable explanation for such declines is that the energy consumption for growth and survival exceeds the carbon fixed by photosynthesis during submergence. On one hand, underwater elongation is an energy-consuming process [18]. Convincing evidence for this is the decline in tuber biomass during the whole submergence (Figure 2C). Although underwater elongation leads to an increase in leaf biomass within a short-term submergence, the decline in leaf biomass within a longer-term submergence is undoubted (Figure 2B). On the other hand, the limited gas diffusion and low light conditions underwater restrict photosynthesis in a submerged plant [7,11,12], even if the plant species has the ability to photosynthesize underwater [11,12,45]. Of course, whether the *A. orientale* plant is capable of underwater photosynthesis still needs further experimental verification. Another reasonable explanation for such declines is acclimation of ROS [15,48], thereby resulting in the death of roots and leaves. Invariably, we observed evident declines in the number of and the biomass of surviving leaves in submerged *A. orientale* plants (Figure 1C and Figure 2B).

Complete submergence can lead to a decline in survival [7,27]. First, both the energy crisis induced by anaerobic metabolism and the accumulation of ROS caused by hypoxia and anoxia reduces plant survival during submergence [15,48]. However, all of *A. orientale* plants survived after each duration of submergence (Table 1), suggesting that *A. orientale* has high flooding tolerance. Second, the post-anoxic injury induced by ROS results in the death of plants during post-submergence [15,48]. For example, 80% of *Chloris gayana* plants survived at the end of 14 d submergence, but subsequently all plants died during post-submergence [43]. As expected, the survival of *A. orientale* plants subjected to 20 d of submergence dropped to 87.5% at the end of recovery growth (Table 1), suggesting that the recovery period is of importance for the survival of *A. orientale*. This is the first study experimentally revealing the resistance of *A. orientale* to complete submergence. Furthermore, the survival rate also is an important criterion influencing the distribution of wild plant species along the altitude gradient in wetlands [11,49]. Many species with high flooding tolerance have high survival after submergence [7,11,49], even after 180 d of submergence [27].

Recovery growth after submergence is of relevance for many plant species [10,20,50]. After a flood recedes, the elongated part of a plant loses support from the water (Figure S5A) and is thereby prone to breakage (Figure S5B) and leaf dehydration (Figure S5C), leading to the death of this part of the plant [6,44,51] (Figure S5B,C). This phenomenon was observed in the *A. orientale* plants during recovery (Figures S3–S5). A series of studies have shown that flood-tolerant plants can regrow rapidly after water recedes [27,50]. In the study, the submerged *A. orientale* plants can generate new leaves within 5 ds after submergence (Figure S4A) and generate more leaves than the controls after submergence, especially after 20 ds of submergence (Figure S4D). The rapid generation of new leaves alleviates the reduction in the leaf number caused by submergence and subsequent post-anoxic injury [49] (Figure S4C,D), as increasing the number of leaves improves both photosynthesis and recovery growth [12,52]. An increasing number of leaves, followed by an increase in leaf length, was observed in the treatment followed by 20 ds of submergence (Figure S4D,L). Although both the total length of surviving leaves and the leaf length increased in the treatment followed by 20 ds of submergence during the whole recovery period, they did not reach the control level (Figure S4D,E,H,L). Submergence can result in the reduction of carbohydrate reserves in the submerged organs [53]. However, a new leaf with low photosynthetic efficiency requires consumption of the plant carbohydrate reserves for rapid growth [52]. Thus, the carbohydrate status of a plant after flooding is a key factor influencing recovery growth and is also recognized as one of the key tolerance criteria [18]. As far as we know, this is the first report focusing on recovery capability and dynamics, an important plant performance criterion for flooding tolerance [20], in *A. orientale*.

One possible caveat of the study is that during the recovery experiments, the end dates of these four submergence treatments for the *A. orientale* plants were inconsistent. These inconsistencies led to differences in climate conditions, especially temperature, which is a key factor for plant growth and development [54], among plants after the four submergence treatments, thereby influencing recovery regrowth during post-submergence. The probably adverse effects of these inconsistencies can be eliminated when the plants treated with different submergence treatments finish simultaneously. Another possible caveat of the study is that the plants used in the study underwent two growing seasons; therefore, the soil on which the plants grew may have become nutrient limited, especially for nitrogen and phosphorus. This may also reduce the differences in recovery growths among these plants, because nutrient application can enhance recovery growth after submergence [55].

Flood events, especially extreme flood events, commonly occur in the Yangtze River basin of China in July and August every year. At the arrival of a great flood peak, complete submergence occurs in the rice cultivated in the lowland paddy fields, resulting in crop yield reduction and economic loss [8,9]. This period is near the end of harvest season for the immature scape and inflorescence of *A. orientale* plants. In other words, most of the immature scapes and inflorescences for *A. orientale* have been gained before flood occurrence. Thus, if the lowland paddy fields could be used to cultivate *A. orientale* plants, the economic loss caused by summer floods for rice may reduce. If *A. orientale* plants were cultivated in the lowland paddy fields during the cultivation season, their plants could be submerged by flash floods, which are often unexpected and uncontrollable, with a depth of up to 50 cm and lasting several days [56] due to extreme precipitation in the context of global warming [2]. The results in the study, however, confirm that *A. orientale* can survive these submergence environments (Figure 1, Figure 2 and Figure 3; Table 1). Thus, the lowland paddy fields can be used to cultivate *A. orientale* to reduce economic loss.

## 4. Materials and Methods

### 4.1. Plant Material

Seeds of *A. orientale* were collected from Yiqian town (116°18′38.13″ E, 26°32′33.8″ N), which has a long cultivation history of *A. orientale*, in Guangchang county, Fuzhou City, Jiangxi Province, China. On the 10 July 2023, seeds were sown in a paddy field in the common garden (116°8′25.3″ E, 29°40′25.2″ N) at Lushan Botanical Garden, Jiangxi Province and Chinese Academy of Science, Jiujiang, China. On the November 1, seedlings with a height of about 10 cm were selected and then transplanted into pots (2 L, one plant per pot) containing mixture with a 5:1 volume ratio of clay to nutritional soil, with a seedling per pot. All of these pots were then put into blue tanks with a length of 62 cm, a width of 38 cm and a height of 15 cm; there were eight plants per tank. All of these tanks were placed on the open field in the common garden to receive full sunlight, and the water level in a tank was not less than a depth of 10 cm during the whole growing season. Entering the winter, the leaves of all plants were withered gradually.

During the spring of 2024, all plants sprouted and grew again. After growing for about 50 days (ds), each of all plants developed three to five fully expanded leaves and grew up to a height of 30 cm. The dead and withered leaves were removed before treatments.

### 4.2. Experiments of Complete Submergence

A total of 64 plants of similar sizes were selected and randomly assigned to two treatments (complete submergence, CS; control, CK), with four durations of treatment (5 ds, 10 ds, 15 ds and 20 ds). The submergence experiments were conducted by using four white plastic tanks (96 cm in diameter and 92 cm in height) filled with tap water that had been aerated, with eight plants per tank. The white plastic tanks were placed in the open field of the common garden. The submergence experiments started on 9 May 2024. The submerged plants all were underwater during the submergence period. The control plants were kept in the blue tanks with a water depth of 10 cm and then placed next to these white plastic tanks.

The leaf number of each plant was recorded at the beginning and at the end of each submergence treatment, and the number of surviving leaves per plant was recorded at the end of each submergence treatment. The lengths of each leaf per plant (the height from soil to the leaf apex) were measured at the end of each submergence treatment. A new leaf was generated when the leaf apex extended out from the leaf sheath (Figure S6). The length of a new leaf was expressed as the length of the penultimate leaf per plant, which is a mature leaf. The total length of the surviving leaves was the sum of the length of the surviving leaves in a plant at the end of submergence. A leaf was considered dead when more than 5/6 of the blade of a leaf had lost its green color (Figure S7A) or a segment of the petiole in a leaf had lost its green color (Figure S7B,C). Eight replicate plants for each treatment were measured. During submergence, *Spirogyra* species were removed from the water in each white tank every day.

After each submergence, the plants were harvested and then divided into roots, leaves and tubers for dry mass determinations for 72 h at 70 °C. The leaf blade was separated from the penultimate leaf in each plant, dried and then weighed. The biomass of surviving leaves was the biomass of the existing green organs still having function. Blade biomass was expressed as the blade biomass of the penultimate leaf per plant. Eight replicate plants for each treatment were measured.

### 4.3. Experiments of Post-Submergence Recovery

A total of 40 plants of similar sizes were selected and randomly assigned to the treatments with five durations of submergence (0 ds, 5 ds, 10 ds, 15 ds and 20 ds). The submergence experiments were conducted by using four white plastic tanks filled with tap water that had been aerated, with eight plants per tank. The white plastic tanks were placed in the open field of the common garden on 19 April 2024. The submerged plants were all underwater during submergence. The plants submerged for 0 d (control) were kept in the blue tanks with a water depth of 10 cm and then placed next to the white plastic tanks. During submergence, *Spirogyra* species were removed from the water in each white tank every day.

At the end of each duration of submergence, a plant was considered dead when the plant had lost green leaves and buds. Subsequently, the remaining plants per treatment were kept in a blue tank with a water depth of 10 cm and then placed in the open field of the common garden. They were used to measure survival and monitor recovery dynamics during the 20 ds recovery periods. A new leaf was generated when the leaf apex extended out from the leaf sheath (Figure S6). During the recovery periods, a plant was considered dead when no new leaf was generated and the old leaves all died; a leaf was considered dead when more than 5/6 of the blade of a leaf had lost its green color (Figure S7).

The survival rate was calculated using the following formula:

$$S=\frac{N}{8}\times 100\%$$

where S and N refer to the survival rate per treatment and the number of surviving plants per treatment, respectively.

During recovery period, the number of new leaves per plant was recorded every day. On days 0, 5, 10, 15 and 20 of the recovery period, the number of surviving leaves was recorded and the length of each surviving leaf per plant was measured. The control plants were continuously monitored for 35 ds. The lengths of time that *A. orientale* plants needed to start to regrow were expressed as the time for generating the first new leaf for each plant during the recovery period. During recovery period, leaf length was expressed as the length of the penultimately surviving leaf per plant; leaf length was expressed as the surviving leaf when the plant had only one leaf. At the end of each recovery treatment, the length of a new leaf was expressed as the length of the penultimately surviving leaf per plant. The total length of the surviving leaves was the sum of the lengths of the surviving leaves in a plant on the day measured.

### 4.4. Data Analyses

Independent-sample *t*-tests were used to compare the differences in the number of new leaves, in the lengths of new leaves, in the number of and total length of surviving leaves, in total biomass, and in the biomass of surviving leaves, of tubers, and of new blades between control and submerged plants during submergence periods. Meanwhile, one-way ANOVA followed by the Duncan’s multiple-range tests were used to compare the differences in the number of new leaves, in the lengths of new leaves, in the number of and total length of surviving leaves, in the total biomass, and in the biomass of surviving leaves, of tubers, and of new blades among plants submerged for four different durations in the submergence experiments. To homogenize the variances, data on the number of new leaves, length of a new leaf, total biomass and biomass of new blades were square-root transformed, and data on the biomass of tubers were cube-root transformed.

Independent-sample *t*-tests were used to compare the differences in the number of new leaves, in the length of new leaves, and in the total length of surviving leaves between the plants submerged and the controls at the ends of the recovery periods. Meanwhile, one-way ANOVA followed by Duncan’s multiple-range tests were used to compare the differences in the lengths of time the plants needed to regrow during the recovery periods and compare the differences in the number of new leaves, in the number of surviving leaves, in the lengths of new leaves, and in the total length of surviving leaves among treatments at the ends of the recovery periods. Mann–Whitney U tests were used to compare the differences in the number of surviving leaves between the plants submerged and the controls at the ends of recovery periods because these data did not meet normality assumptions and equal variance, even after data transformation.

Data sets on recovery dynamics for the *A. orientale* plants did not meet normality assumptions and equal variance, even after data transformation. Thus, Mann–Whitney U tests were used to compare the differences in number of and total length of surviving leaves and on leaf length between non-submerged and submerged plants during recovery periods. All analyses were performed in SPSS 20.0 (IBM, Armonk, NY, USA).

## 5. Conclusions

The present findings have shown that *A. orientale* plants responded to complete submergence by leaf elongation, had high survival rates, and reached the level of the control plants rapidly within 20 ds of recovery time, indicating that *A. orientale* can survive the complete submergence caused by flooding and resume growth rapidly after the deluge. The obtained results add to the growing literature on the mechanisms of flooding tolerance in wetland plants and crops and provide valuable information to improve the flooding tolerance of *A. orientale* plants, an important herb with medicinal value and edibility. *A. orientale* plants, however, will be imposed upon by greater frequencies of flooding and higher flood peaks caused by intense precipitation in the era of global climate change. Whether and how submergence depths and frequencies influence the survival and fitness of *A. orientale* plants is needed to be studied and make further research imperative.

## Figures and Tables

**Table 1:** The survival rate of *Alisma orientale* (Samuel.) Juz.plants at the ends of the submergence and recovery periods.

Periods	Duration of Submergence (ds)				
	0	5	10	15	20
At the end of submergence	100%	100%	100%	100%	100%
At the end of recovery	100%	100%	100%	100%	87.5%

## Data Availability

Data are contained within the article and Supplementary Materials.

## Supplementary Materials

The following supporting information can be downloaded at: https://www.mdpi.com/article/10.3390/plants13223189/s1, Figure S1: The structure for a *Alisma orientale* plant, its medicinal tuber and edible scape and inflorescence. Figure S2: Submergence-induced petiole elongation in an *A. orientale* plant. Figure S3: Plant performance at 0 d and 3 d of recovery for *A. orientale* plants submerged for 5 ds. Figure S4: Recovery growth dynamics of *A. orientale* plants after complete submergence. Figure S5: Plant performance for *A. orientale* after complete submergence. Figure S6: A new leaf occurs for *A. orientale* plant. Figure S7: A leaf was considered dead.
