# Supplementary material for: Effects of Complete Submergence on Growth, Survival and Recovery Growth of Alisma orientale (Samuel.) Juz
Source: Plants (Basel). 2024 Nov 13;13(22):3189. doi: 10.3390/plants13223189 (PMC11598203; doi:10.3390/plants13223189)
Supplement: Supplementary file 1 [file plants-13-03189-s001.zip › plants-3263268-supplementary.pdf]

Supplementary Materials:

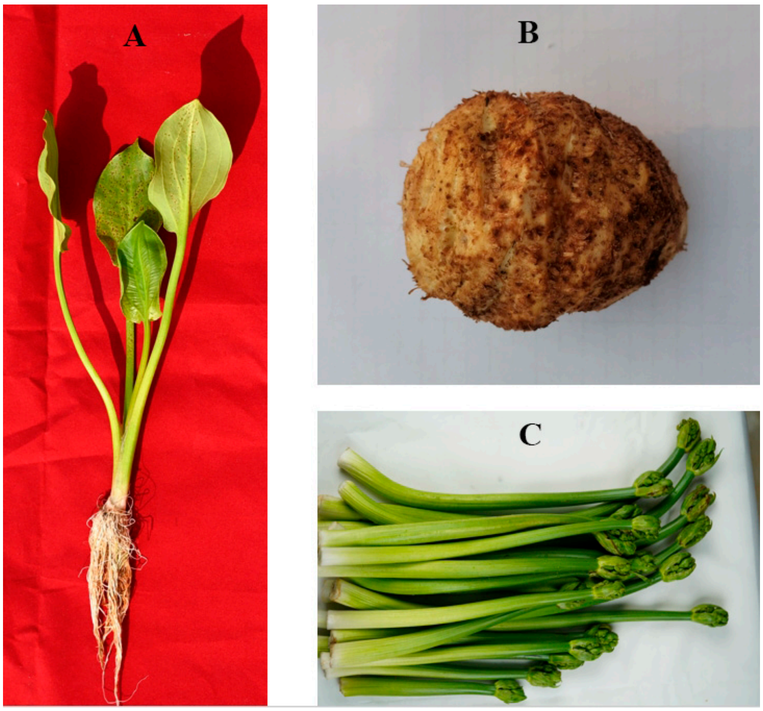

Figure S1. The structure for a *Alisma orientale* (Samuel.) Juz. plant (**A**), its medicinal tuber (**B**), and edible scape and inflorescence (**C**).

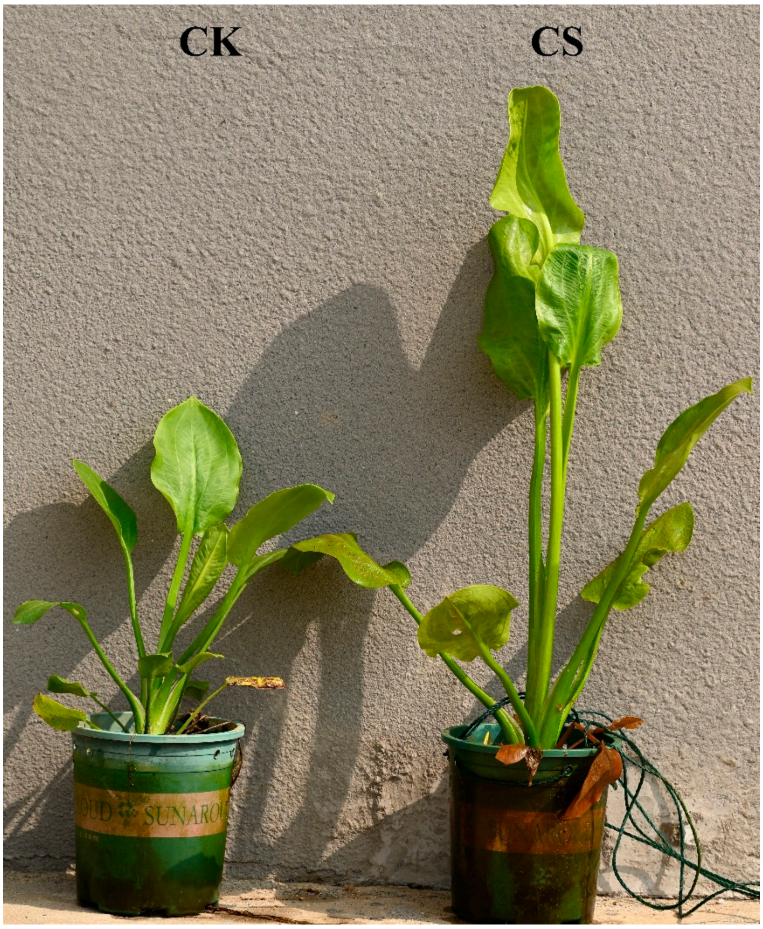

Figure S2. Submergence-induced petiole elongation in a *A. orientale* plant. CK, control; CS, complete submergence.

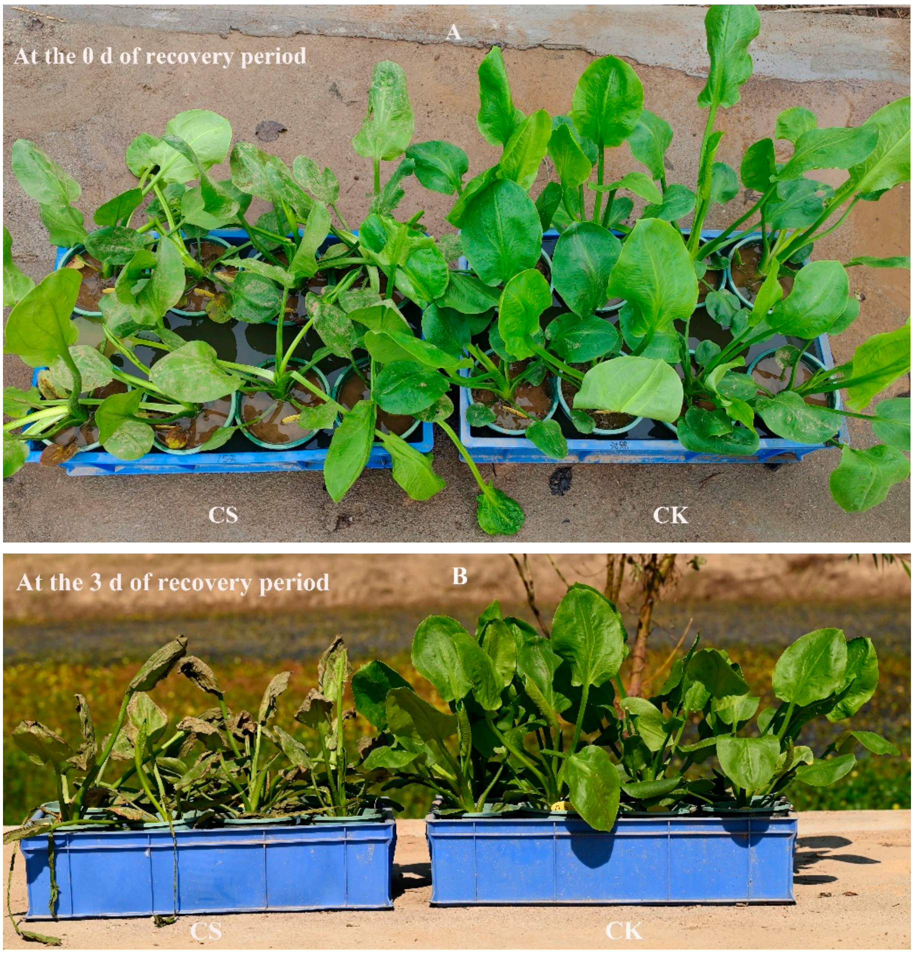

Figure S3. Plant performance at the 0 d of and at the 3 d of recovery period for *A. orientale* plants submerged for 5 ds. (**A**) at the 0 d of recovery period; (**B**) at the 0 d of recovery period. CK, control; CS, complete submergence.

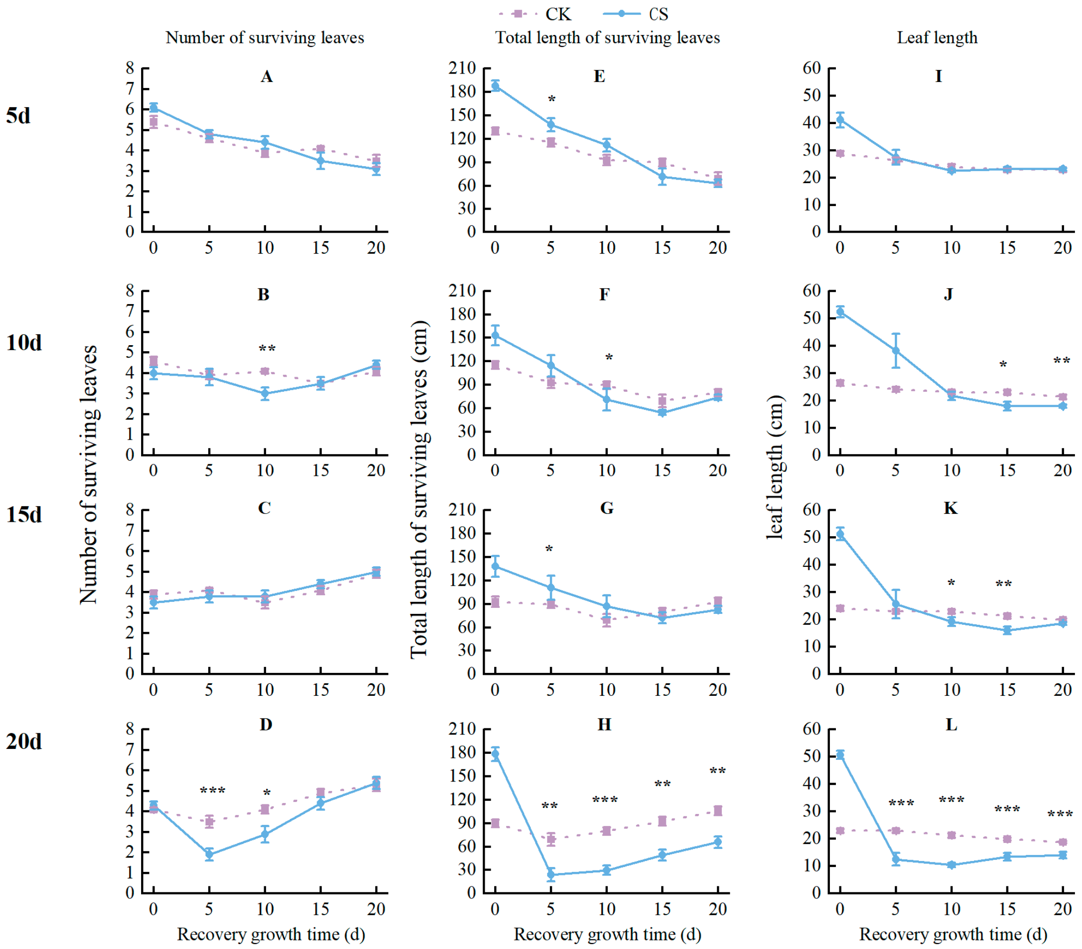

Figure S4. Recovery growth dynamics of *A. orientale* plants after complete submergence. Leaf length was expressed as the length of the penultimately surviving leaf per plant; leaf length was expressed as the surviving leaf when the plant had only one leaf. CK, control; CS, complete submergence. *, $P < 0.05$; **, $P < 0.01$; ***, $P < 0.001$; Mann-Whitney U tests. Data are means ± SE. Number of replicates is eight, except for the plants submerged for 20 d (n = 7).

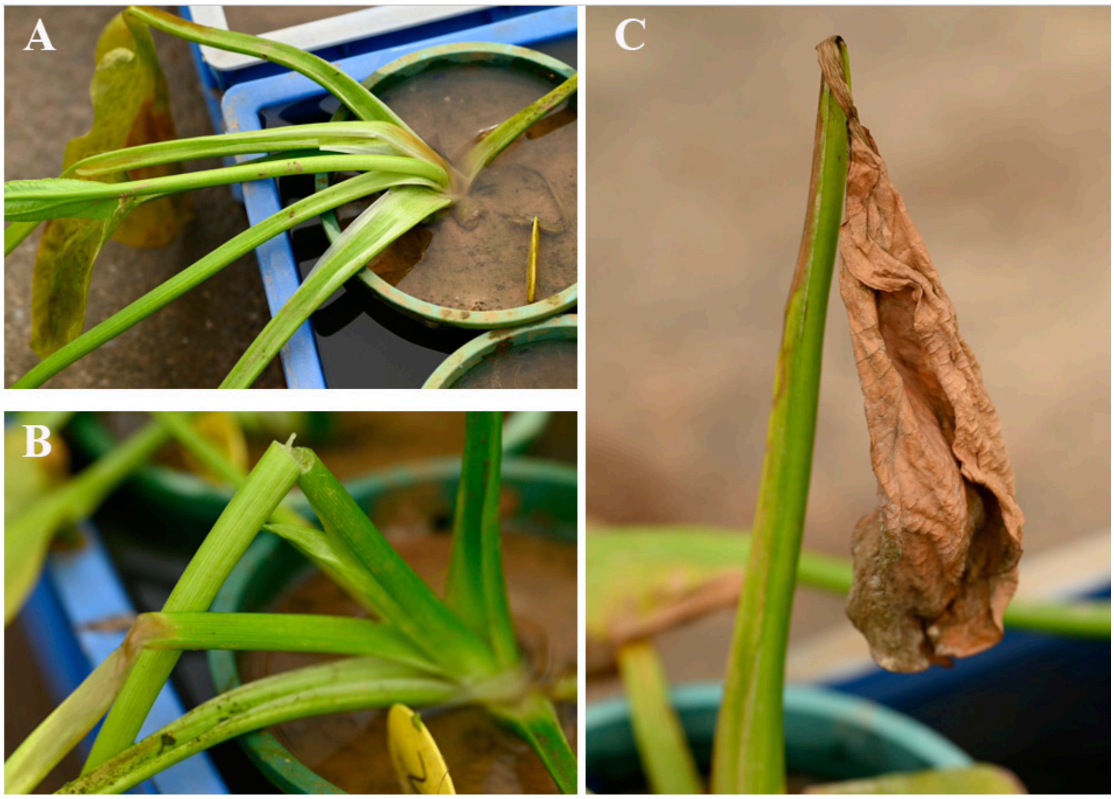

Figure S5. Plant performance for *A. orientale* after complete submergence. (**A**) without-supported leaves for *A. orientale* after submergence. (**B**) An easily-broken petiole for *A. orientale* after submergence. (**C**) A dehydration leaf for *A. orientale* at the 5 ds of recovery period.

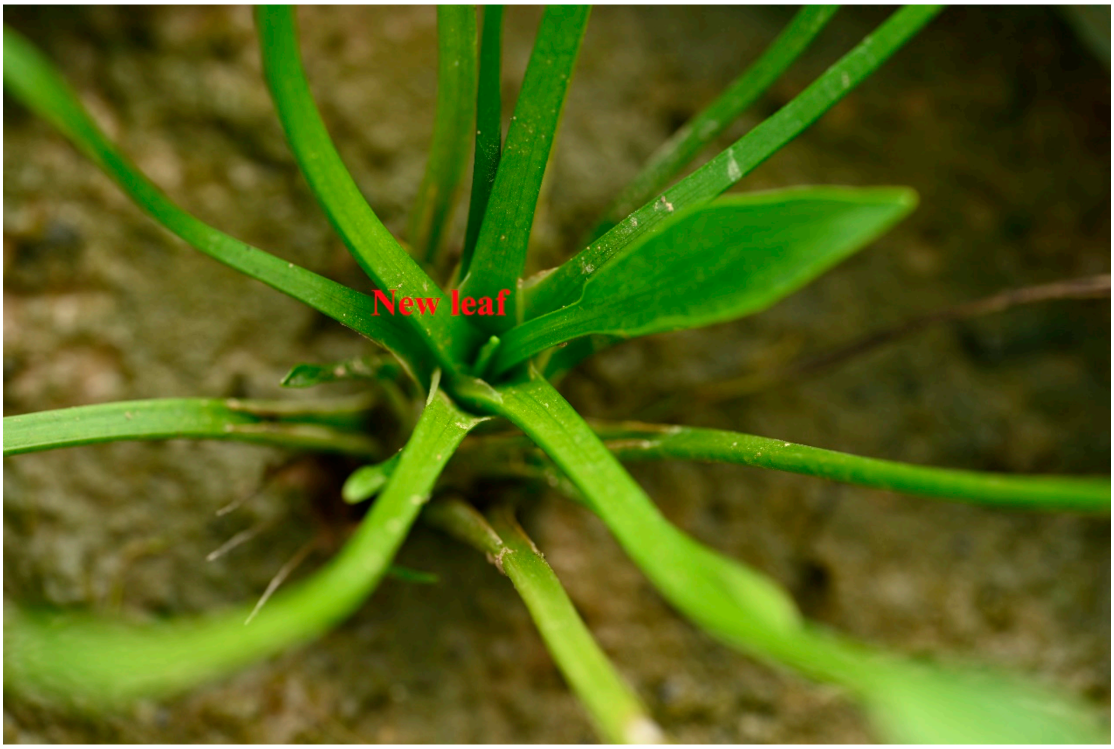

Figure S6. A new leaf occurs for *A. orientale* plant.

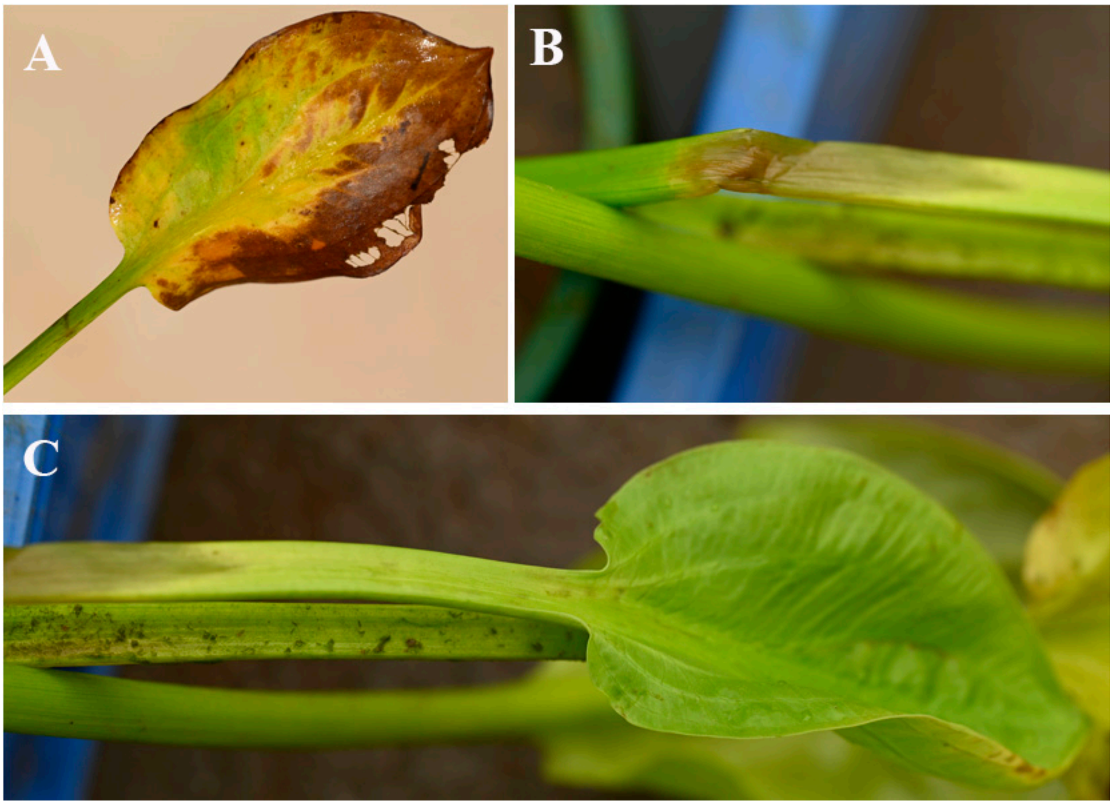

Figure S7. A leaf was considered dead. (**A**) more than 5/6 of the blade in a leaf lost green. (**B**) a segment of the petiole in a leaf lost green. (**C**) A leaf with dead segment of petiole.
